# Optimizing Workflows for Fast and Reliable Metabolic Tumor Volume Measurements in Diffuse Large B Cell Lymphoma

**DOI:** 10.1007/s11307-020-01474-z

**Published:** 2020-01-28

**Authors:** Coreline N. Burggraaff, Fareen Rahman, Isabelle Kaßner, Simone Pieplenbosch, Sally F. Barrington, Yvonne W.S. Jauw, Gerben J.C. Zwezerijnen, Stefan Müller, Otto S. Hoekstra, Josée M. Zijlstra, Henrica C.W. De Vet, Ronald Boellaard, On behalf of the PETRA Consortium

**Affiliations:** 1 Department of Hematology, Cancer Center Amsterdam, Amsterdam UMC, Vrije Universiteit Amsterdam, De Boelelaan 1117, 1081HV Amsterdam, Netherlands; 2 Department of Clinical Oncology, Guy’s and St Thomas’ NHS Foundation Trust, Guy’s Cancer, London Bridge, London, UK; 3 Department of Nuclear Medicine, University Hospital Essen, University of Duisburg-Essen, Forsthausweg 2, 47057 Duisburg, Germany; 4 King’s College London and Guy’s and St Thomas’ PET Centre, School of Biomedical Engineering and Imaging Sciences, King’s Health Partners, King’s College London, London, UK; 5 Department of Radiology and Nuclear Medicine, Cancer Center Amsterdam, Amsterdam UMC, Vrije Universiteit Amsterdam, De Boelelaan 1117, 1081HV Amsterdam, Netherlands; 6 Department of Epidemiology and Biostatistics, Amsterdam Public Health Research Institute, Amsterdam UMC, Vrije Universiteit Amsterdam, De Boelelaan 1105, 1081HV Amsterdam, Netherlands

**Keywords:** Diffuse large B cell lymphoma, Metabolic tumor volume, PET/CT, Total lesion glycolysis

## Abstract

**Purpose:**

This pilot study aimed to determine interobserver reliability and ease of use of three workflows for measuring metabolic tumor volume (MTV) and total lesion glycolysis (TLG) in diffuse large B cell lymphoma (DLBCL).

**Procedures:**

Twelve baseline [^18^F]FDG PET/CT scans from DLBCL patients with wide variation in number and size of involved organs and lymph nodes were selected from the international PETRA consortium database. Three observers analyzed scans using three workflows. Workflow A: user-defined selection of individual lesions followed by four automated segmentations (41%SUVmax, A50%SUVpeak, SUV≥2.5, SUV≥4.0). For each lesion, observers indicated their “preferred segmentation.” Individually selected lesions were summed to yield total MTV and TLG. Workflow B: fully automated preselection of [^18^F]FDG-avid structures (SUV≥4.0 and volume≥3ml), followed by removing non-tumor regions with single mouse clicks. Workflow C: preselected volumes based on Workflow B modified by manually adding lesions or removing physiological uptake, subsequently checked by experienced nuclear medicine physicians. Workflow C was performed 3 months later to avoid recall bias from the initial Workflow B analysis. Interobserver reliability was expressed as intraclass correlation coefficients (ICC).

**Results:**

Highest interobserver reliability in Workflow A was found for SUV≥2.5 and SUV≥4.0 methods (ICCs for MTV 0.96 and 0.94, respectively). SUV≥4.0 and A50%Peak were most and SUV≥2.5 was the least preferred segmentation method. Workflow B had an excellent interobserver reliability (ICC = 1.00) for MTV and TLG. Workflow C reduced the ICC for MTV and TLG to 0.92 and 0.97, respectively. Mean workflow analysis time per scan was 29, 7, and 22 min for A, B, and C, respectively.

**Conclusions:**

Improved interobserver reliability and ease of use occurred using fully automated preselection (using SUV≥4.0 and volume≥3ml, Workflow B) compared with individual lesion selection by observers (Workflow A). Subsequent manual modification was necessary for some patients but reduced interobserver reliability which may need to be balanced against potential improvement on prognostic accuracy.

**Electronic supplementary material:**

The online version of this article (10.1007/s11307-020-01474-z) contains supplementary material, which is available to authorized users.

## Introduction

In young patients with diffuse large B cell lymphoma (DLBCL), a large maximum tumor diameter is an indicator of poor prognosis [1]. Recent progress in lymphoma care has recommended exploration of the prognostic value of volumetric tumor bulk measured on staging 2-deoxy-2-[^18^F]fluoro-d-glucose ([^18^F]FDG) PET/CT, with methods combining metabolic activity and volume [2]. In lung cancer patients, studies have focused on finding the most reliable tumor segmentation method [3–5]. However, compared with lung cancer, lymphoma segmentation is more challenging due to higher number of lesions, multiple anatomical locations, and inter- and intratumoral [^18^F]FDG uptake heterogeneity.

Preliminary data suggest that baseline metabolic tumor volume (MTV) has a prognostic value in DLBCL [6–9] and predict outcome better than bulky disease measured by maximum tumor diameter [7]. Total lesion glycolysis (TLG)—defined as SUVmean in a volume multiplied by the corresponding MTV—seems to perform similarly [7] or inferiorly [6, 8] in predicting outcome of DLBCL patients. Various segmentation methods to measure MTV and TLG are being used in clinical lymphoma studies [10]: most use a fixed SUV threshold (*e.g.,* SUV≥2.5 [7, 9] or SUV≥4.0 [11]) or a percentage of SUVmax (*e.g.,* 41 % of SUVmax [6, 8, 12]) to define MTV. An important finding from earlier studies in DLBCL is that optimal cutoff values range widely (220–550 ml), probably because of using different methodologies, small patient cohorts, differences in patient risk factors, and therapies [13]. Moreover, these data-driven cutoff values should be interpreted with caution, as they depend highly on acquisition and reconstruction protocols.

Segmentation methods in these studies are generally derived from phantom experiments [4, 12], or correlation with pathological specimens in lung cancer [4]. Limited data are available about the differences in ease of use in the lymphoma clinical setting and interobserver reliability of these tumor segmentation methods [10]. Previous studies in DLBCL [10], T cell [14], and Hodgkin lymphoma [15] showed that different segmentation methods, despite having different cutoff values, show comparable accuracy for predicting survival. Therefore, for future use in practice and clinical trials a robust, reliable and easy—*i.e.,* with least required observer interaction—segmentation workflow is necessary. To the best of our knowledge, this is the first pilot study in DLBCL that compares interobserver reliability and ease of use of three workflows for measuring MTV and TLG, and that assesses the effect of manual modification on interobserver reliability.

## Materials and Methods

### Study Population

Twelve baseline [^18^F]FDG PET/CT scans from newly diagnosed DLBCL patients with wide variation in number and size of involved organs and lymph nodes lesions were selected from the international PETRA database (http://www.petralymphoma.org). The use of all data within the PETRA imaging database has been approved by the Medical Ethics Review Committee of the VU University Medical Center (JR/20140414) after patients’ consent to participate in the studies included in the database.

### Image Analysis Workflows A and B

Two semi-automated workflows (Workflows A and B) were performed in the same week, by three independent observers using the ACCURATE software tool [16]. Manual modifications of the semi-automatically generated volumes of interest (VOIs) were not allowed initially. The workflow with the best interobserver reliability and ease of use was selected as starting point for manual modification in Workflow C.

Workflow A comprised a user-defined selection of individual lesions. The observers had to select individual lesions (by a single mouse click in the “hottest” part of each lesion), followed by automated segmentation in the tool using four separate frequently published segmentation methods:

1. 41 % of SUVmax (41%MAX)
2. A50% of SUVpeak, *i.e.,* 50 % of SUVpeak with local background correction [17] (A50%P)
3. fixed SUV threshold of 2.5 (SUV≥2.5)
4. fixed SUV threshold of 4.0 (SUV≥4.0).

The four segmentation methods were initiated from one single click by the observer, to avoid introduction of extra variability by repeated clicking. Moreover, the tool first calculated a robust local maximum (using a region growing method applying a 70 % threshold of the point clicked) in order to be less dependent on the exact point clicked by the observer. Generated VOIs were summed for all lesions selected by each observer to calculate MTV and TLG according to each of the four segmentation methods.

To explore the use and performance of consensus methods, two methods were added afterwards, which use the delineations found with the above four standard methods as input for a majority vote (MV) approach [18]. MV volumes were defined by all voxels included in the MTV or TLG by at least two (MV2) or three (MV3) of the input methods.

Workflow B consisted of a fully automated preselection of [^18^F]FDG-avid structures defined by an SUV≥4.0 and a volume threshold of ≥3 ml. These preselected regions resulted into an identical starting point for all observers but could include non-tumor regions with normal increased [^18^F]FDG uptake, such as the brain or bladder. From this starting point, the observers decided on the removal of non-tumor regions by using a clearing option (*i.e.,* single click(s)) or spatial limits to reduce the analyzed field of view (*e.g.,* using a slider option to exclude superior slices including the brain or inferior slices including the bladder); after this, only lymphoma lesions remain. Therefore, a region is defined as any preselected 3D-VOI with uptake above the SUV≥4.0 threshold, whereas a lesion is defined as a 3D-VOI identified by the observer as lymphoma.

To determine ease of use for both workflows, each observer noted the total analysis time per patient (including loading of the scan, performing the analysis, and saving results).

In addition, the success of all semi-automatically generated VOIs was rated by each observer according to the following definitions:

- *Failed: generated VOI is unrealistic or does not contain complete lesion*
- *Poor: generated VOI takes into account physiological uptake or contains a lot of background and manual modification is needed*
- *Acceptable: only minimal manual modification needed for good VOI*
- *Good: generated VOI is comparable to what you consider to be lymphoma*

A mean “success rate” (all acceptable and good ratings) was calculated for each method. Finally, observers had to choose one “preferred segmentation” for the generated VOIs. The MV2 and MV3 consensus methods were rated by one experienced observer according to the same success definitions. As these MV methods were assessed afterwards, they could not be chosen as “preferred segmentation.”

### Image Analysis Workflow C

The observers used the fully automated method as in Workflow B for the analyses on the same twelve scans (Workflow C1). These analyses were performed 3 months later to minimize recall bias. In addition to the interactive deletion of physiological uptake regions similar to Workflow B, the observers were allowed in Workflow C to manually modify the generated VOIs by adding missed lesions (with the A50%P option or manually) and removing of physiological uptake with an “eraser” tool. The manually modified MTVs and TLGs were checked for correct delineation and identification of tumor sites (and changed if needed) by independent nuclear medicine physicians (NM, one per observer) with more than 10 years of experience with [^18^F]FDG PET/CT evaluation in lymphoma (OSH, SFB, SM; Workflow C2).

### Statistical Analysis

Success rates of generated VOIs were analyzed descriptively. Interobserver reliability was expressed as intraclass correlation coefficients (ICCs) and coefficients of variation (CoVs). ICC estimates and their 95 % confidence intervals (95%CIs) were calculated with a two-way random-effects model for absolute agreement [19]. The 95%CIs of the ICC values were interpreted as poor (< 0.5), moderate (0.5–0.75), good (0.75–0.9), and excellent (> 0.9) [20, 21]. CoV was calculated as the ratio of the standard deviation (over three observers) of MTVs or TLGs divided by the mean values per patient. Mean CoVs are presented, *i.e.,* CoVs averaged over all patients. Bland-Altman plots were drawn to visually assess potential bias of the mean differences between the workflows and to estimate 95 % limits of agreement [22]. Normality of MTV and TLG differences before and after manual modification was checked with the Shapiro-Wilkinson (SW) test, in which *P* < 0.05 was an indication of a non-normal distribution. Statistical analyses were performed using SPSS Statistics (IBM, v.20).

## Results

### Workflow A; Individual Lesion Selection

#### Lesion Selection

The total number of selected lesions for observer 1, 2, and 3 was 162, 117, and 118, respectively, which was due to the fact that observer 1 separately selected small lesions close to larger lesions, which were ignored by observers 2 and 3. It resulted in larger volumes for the A50%P and the 2 MV consensus methods for observer 1 (Supplemental Fig. 1). In total, 76 lesions were selected by all observers; of which, 35 showed identical segmentation results, and 18 lesions had a difference in volume between observers of < 1 ml. Twenty-three non-identical lesions were caused by clicking in different parts of a heterogeneous lesion, which resulted in missing the SUVmax or SUVpeak of the lesion.

#### Interobserver Reliability

ICC values for semi-automated MTVs were 0.43, 0.86, 0.96, and 0.94 for the 41%MAX, A50%P, SUV≥2.5, and SUV≥4.0 thresholds, respectively. Mean CoVs were 65.5 %, 36.7 %, 13.3 %, and 13.8 %, respectively (Table 1). When considering the 95%CIs of ICCs, only SUV≥2.5 and SUV≥4.0 showed excellent and good to excellent reliability, respectively.

**Table 1.** Interobserver reliability of semi-automated MTV and TLG assessment for the different workflows

	MTV			TLG		
	Mean(range)	Mean CoV(range)	ICC(95%CI)	Mean(range)	Mean CoV(range)	ICC(95%CI)
Workflow A (individual lesion selection)						
41%MAX	1106(33–4991)	65.54(0–164.38)	0.43(0.08–0.76)	6236(471–21,431)	54.57(0–151.84)	0.37(0.02–0.72)
A50%P	550(34–4153)	36.74(0–139.73)	0.86(0.68–0.95)	5736(245–45,441)	26.76(0–118.26)	0.93(0.82–0.98)
SUV≥2.5	2399(73–7404)	13.34(0–54.21)	0.96(0.91–0.99)	15,902(347–55,588)	7.11(0–33.81)	0.99(0.98–1.00)
SUV≥4.0	1289(30–5688)	13.78(0–83.59)	0.94(0.86–0.98)	13,617(220–50,068)	11.32(0–82.52)	0.97(0.93–0.99)
MV2	1505(59–6258)	22.68(0–83.59)	0.92(0.80–0.97)	14,422(301–51,908)	15.84(0–82.52)	0.97(0.91–0.99)
MV3	927(33–4654)	33.54(0–154.17)	0.91(0.79–0.97)	12,181(229–43,669)	24.91(0–135.92)	0.96(0.89–0.99)
Workflow B (automated preselection)						
SUV≥4.0, Volume≥3ml	1004(23–5723)	2.32(0–10.43)	1.00(1.00–1.00)	8446(189–50,779)	1.85(0–7.49)	1.00(1.00–1.00)
Workflow C (automated preselection with manual modification)						
Final MTV	1115(53–5589)	16.71(0–109.46)	0.92(0.82–0.98)	8610(284–48,079)	13.33(0–111.83)	0.97(0.93–0.99)

*MV*, majority vote; *MTV*, metabolic tumor volume; *CoV*, coefficient of variation; *ICC*, intraclass correlation coefficient; *CI*, confidence interval; *TLG*, total lesion glycolysis

For the MV2 and MV3 consensus methods, the mean CoVs were 22.7 % and 33.5 % and ICCs were 0.92 and 0.91, respectively. Overall, fixed SUV threshold methods (SUV≥2.5 and SUV≥4.0) showed least interobserver variability for MTV assessment in Workflow A. TLG showed similar ICCs and CoVs for these two methods.

#### Ease of Use

Mean analysis time in Workflow A was 28.7 min per patient (range 5–63, Table 2). The most preferred method differed per patient and between observers (Table 3). A50%P and SUV≥4.0 were most often chosen as “preferred segmentation” on a patient-level with success rates (rated as acceptable or good segmentations of visible tumor) ranging from 33 to 87 % and 35–76 %, respectively. The mean success rate for the 41%MAX method ranged from 31 to 86 % between observers. The success rates for the MV2 and MV3 methods, as scored by one observer, were 84 % and 87 %, respectively. Although SUV≥2.5 showed the highest observer reliability, this method was chosen only in 2 patients as the most preferred method by 1 observer. The mean success rate for this method ranged between 27 and 39 % between observers. This method tended to overestimate the tumor volumes (Supplemental Figs. 1–2). Therefore, we decided to focus on the SUV≥4.0 method as preselection criterion.

**Table 2.** Mean analysis time for the different workflows in minutes (mean ± standard deviation (range))

Workflow	A individual lesion selection (*n* = 12)	B automated preselection (*n* = 12)	C with manual modification (*n* = 12)
Observer 1	29.1 ± 20.8(5–63)	7.2 ± 3.7(3–15)	23.3 ± 13.4(5–45)
Observer 2	Not reported*	Not reported*	26.7 ± 15.6(10-62)
Observer 3	28.2 ± 13.7(15–60)	7.3 ± 3.5(1–12)	16.7 ± 9.7(8–42)
Mean	28.7^†^	7.3^†^	22.2

*Observer 2 summed the total time for Workflow A + B; mean 27.3 ± 19.2 (7–75) minutes

^†^Mean value based on 2 observers

**Table 3.** Most preferred method per observer for Workflow A

Patient	Observer 1	Observer 2	Observer 3
1	41%MAX	41%MAX	SUV≥4.0
2	41%MAX	41%MAX/A50%P/SUV≥4.0	SUV≥2.5
3	A50%P	41%MAX	SUV≥4.0
4	SUV≥4.0	A50%P	SUV≥4.0
5	SUV≥4.0	A50%P	SUV≥4.0
6	SUV≥4.0	41%MAX/A50%P	SUV≥4.0
7	A50%P	A50%P	SUV≥2.5
8	A50%P	41%MAX/A50%P	SUV≥4.0
9	A50%P	41%MAX	SUV≥4.0
10	A50%P	A50%P	A50%P
11	SUV≥4.0	41%MAX	A50%P
12	41%MAX/SUV≥4.0	41%MAX	A50%P

Each observer indicated their “preferred segmentation” for individual lesions. The most preferred method per patient was defined as the method most often noted as “preferred segmentation”

### Workflow B; Preselection Strategy

#### Lesion Selection

The total number of selected tumor regions for observer 1, 2, and 3 was 76, 76, and 77, respectively.

Seventy-two identical tumor regions were selected by all three observers.

#### Interobserver Reliability

**Fig. 1.**
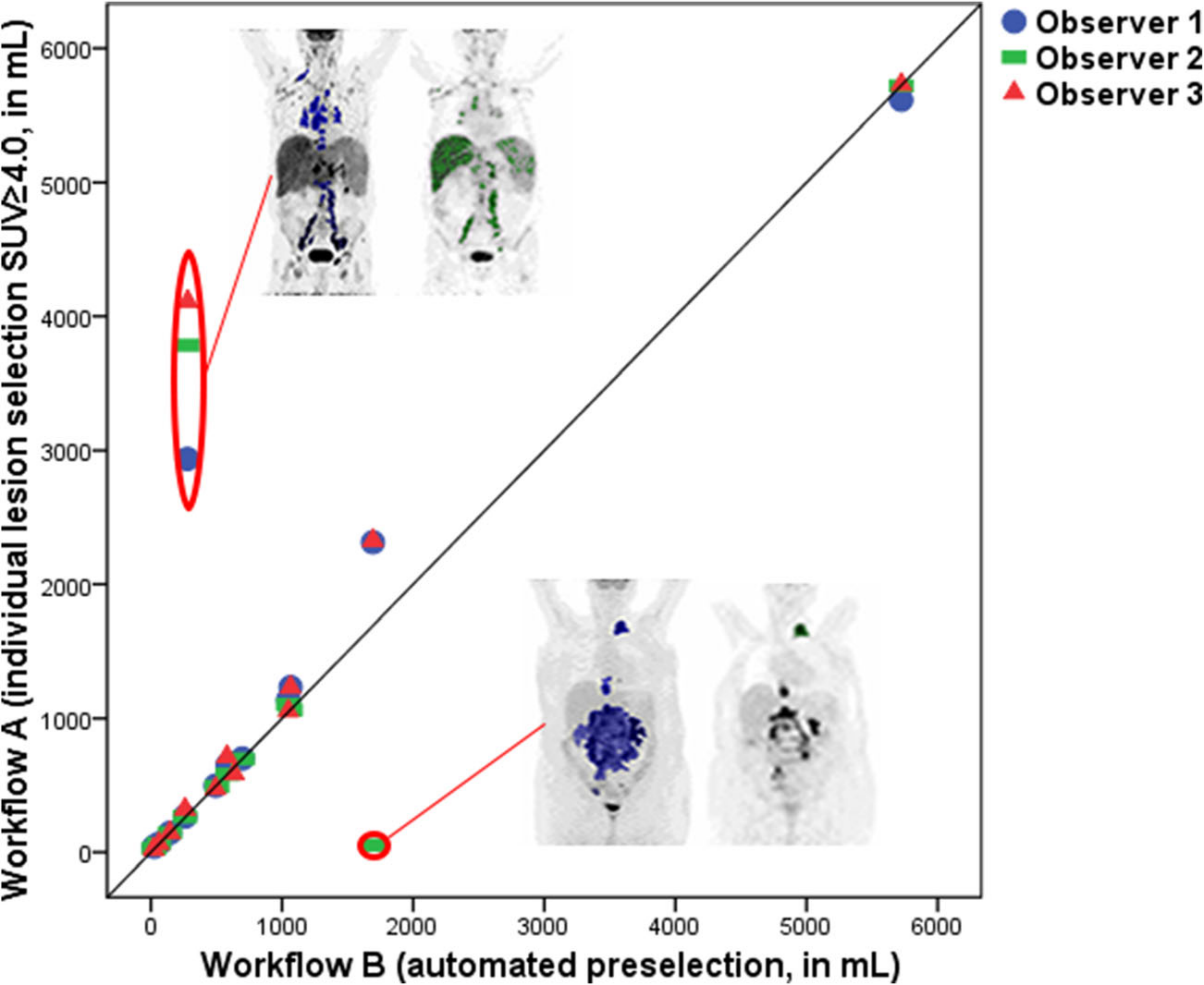
Scatterplot of MTV for Workflow A (user-defined selection with SUV≥4.0) and Workflow B (automated preselection). PET images represent examples of different MTV interpretations between the workflows. Top left images (patient 10): Workflow B contains only lymphoma lesions around the large vessels (left), while in Workflow A, the liver and spleen were also included in the lesion selection (right). Bottom right images (patient 8): in Workflow B, the large lesion was selected (left), while it was interpreted as not being lymphoma in Workflow A (right).

Workflow B is based on the SUV≥4.0 threshold and showed good correlation with SUV≥4.0 threshold of Workflow A with a Pearson correlation of 0.812 (after removing 4 volumes as outliers in 2 patients 0.995, Fig. 1). Outliers were caused by one patient with many lesions, in whom the SUV≥4.0 threshold failed (large parts of the liver and spleen were included in this segmentation) and another with a large abdominal lesion that was interpreted as non-lymphoma by one observer. Complete agreement of the preselected volumes on a patient-level between all observers was found in six patients. The ICC value for generated MTVs in this workflow was excellent (1.00, 95%CI 1.00–1.00) and the mean CoV was 2.3 % (range 0–10.4 %, Table 1), with similar results for TLG.

#### Ease of Use

Time to complete Workflow B ranged from 1 to 15 min (mean 7.3, Table 2). Preselected MTVs were rated as successful in seven, three, and four patients by the observers, respectively. They were classified as failures in zero, four, and six patients respectively.

### Workflow C; Manual Modification

#### Effect of Manual Modification

After manual modification of the preselected volumes the ICC of the final MTV was 0.92 (95%CI 0.82–0.98, Table 1). Mean CoV for the final MTV was 16.7 %. Results for TLG again were similar, with excellent ICC values and good to excellent ICC values for MTV. The total time to perform this workflow ranged from 5 to 62 min (mean 22.2, Table 2).

**Fig. 2.**
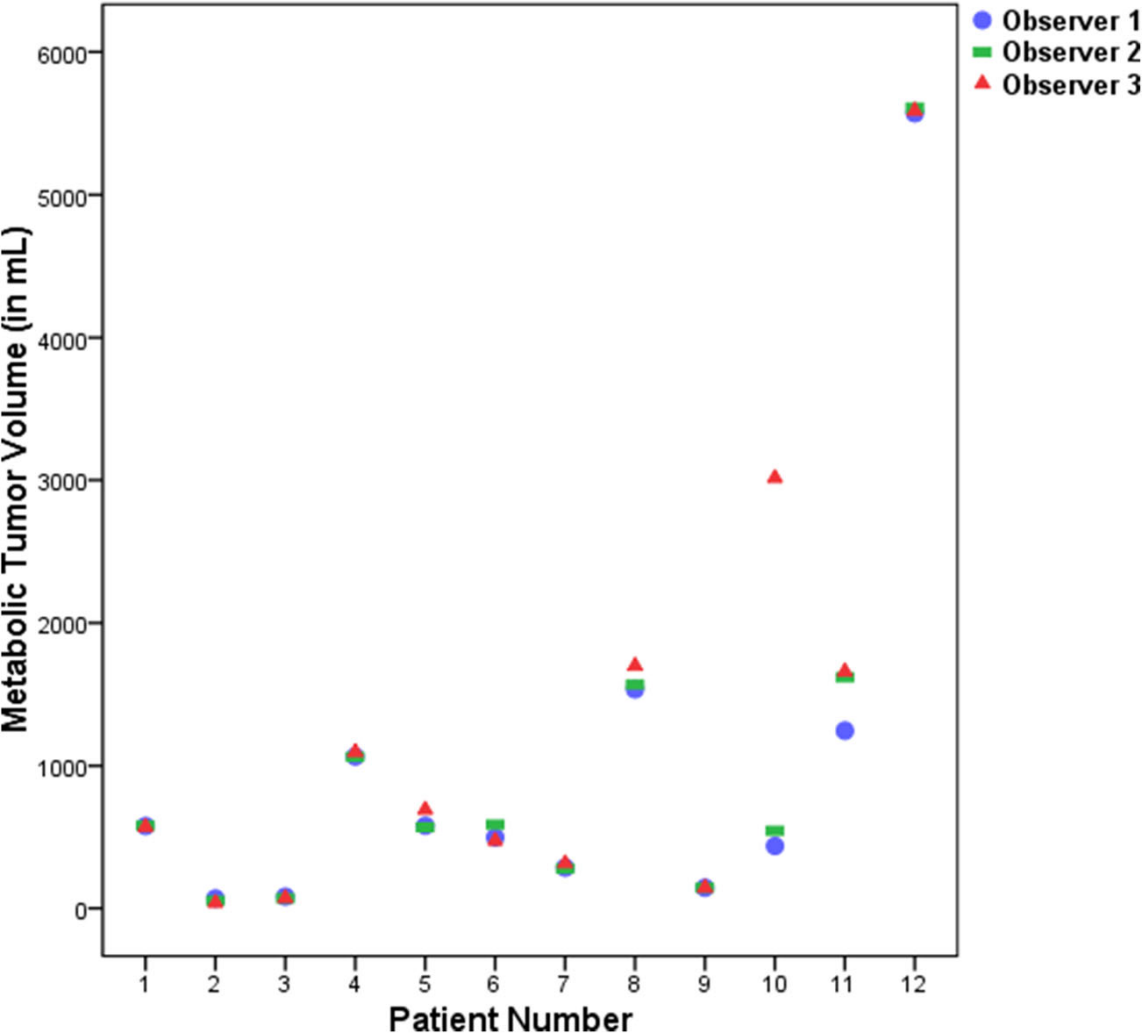
Scatterplot of final MTV assessment in Workflow C.

**Fig. 3.**
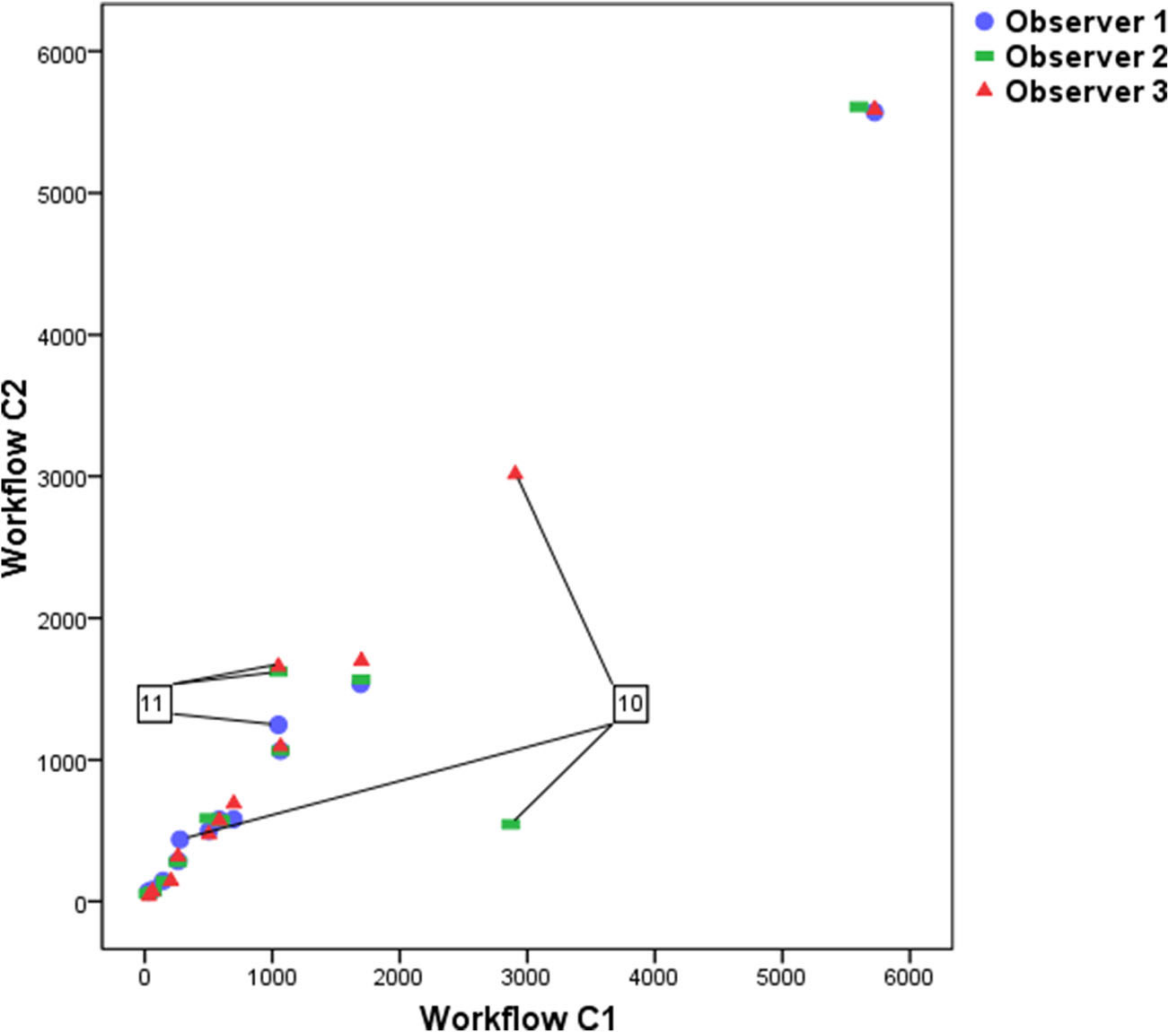
Scatterplot of MTV assessment in Workflow C (automated preselection before (C1)—and final MTV after manual modification (C2), in milliliters). Datapoints from two challenging patients (patients 10 and 11) are indicated by lines. The numbers in the boxes refer to the patient numbers described in the main text.

**Fig. 4.**
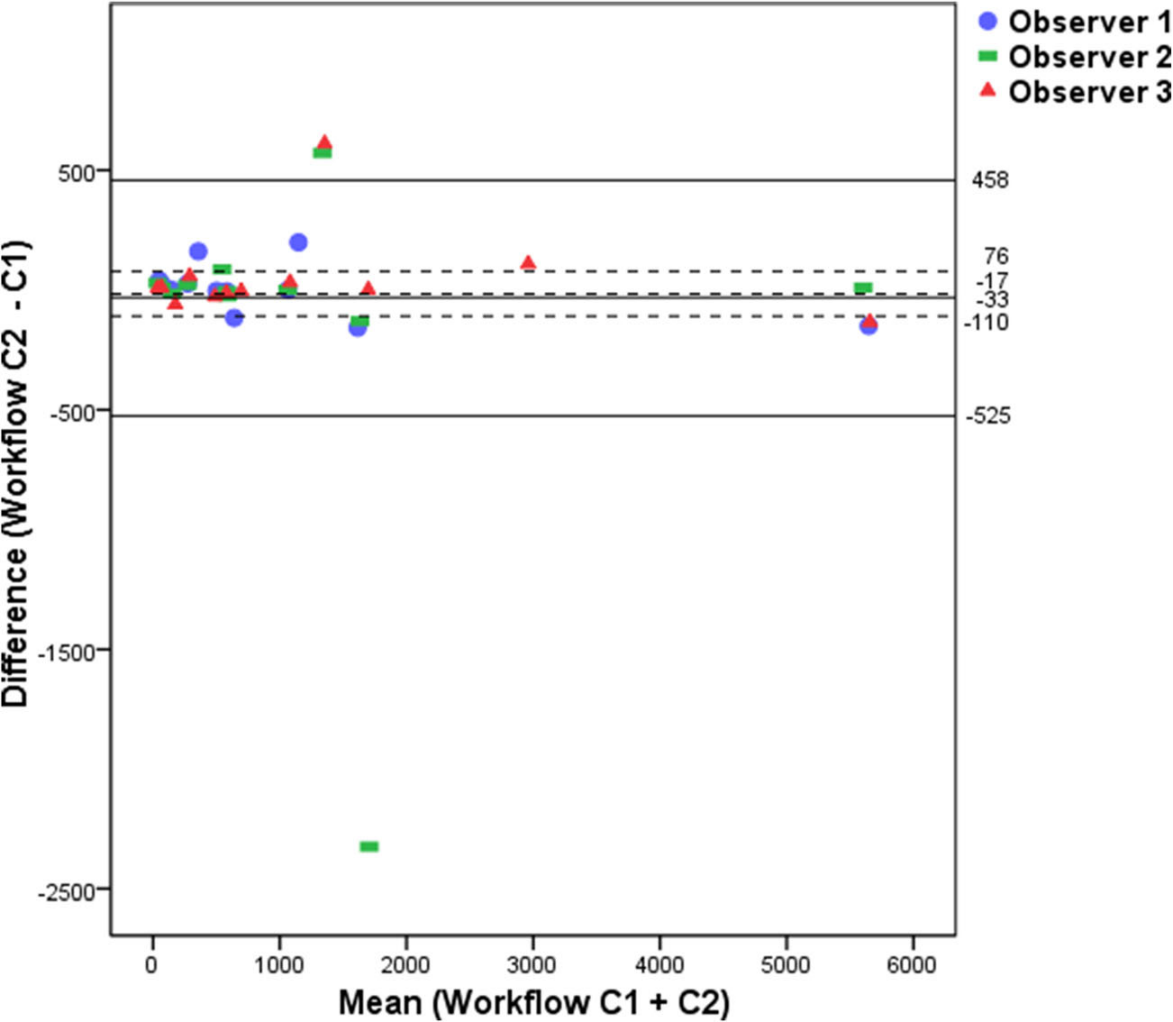
Bland-Altman plot showing effect of manual modification of MTV assessment in Workflow C (automated preselection before (C1)—and final MTV after manual modification (C2)). Solid line: mean value, upper- and lower limit of agreement without exclusion of outliers. Dashed line: mean value, upper- and lower limit of agreement after exclusion of patients 10 and 11.

Figure 2 shows the modified MTVs approved by a nuclear medicine physician (final MTV). Figure 3 shows a scatterplot of the correlation between the preselected and final MTV in Workflow C. Interestingly, the same outlier (patient 10) occurred as in Fig. 1, but contrary to this, two observers now decided to keep the entire liver in the preselection of Workflow C while they removed the liver uptake in Workflow B. For the final MTV, observer 2 had to remove the liver uptake after the check by the experienced NM physician. In another patient (patient 11), the preselection missed many small bone lesions, which were added manually. Figure 4 shows the Bland-Altman plot of the preselected and final MTV in Workflow C. The 95 % limits of agreement ranged widely (− 525 to 458). The differences between preselected and final MTV did not have a normal distribution according to the SW test (*P* = 0.002). After excluding patients 10 and 11 (Figs. 3 and 4) described as outliers, the mean difference had a normal distribution (*P* = 0.106). The plot shows both the original—as well as the recalculated 95 % limits of agreement after exclusion of the outliers.

## Discussion

We assessed the interobserver reliability and ease of use of three workflows for measuring MTV and TLG in 12 DLBCL patients and found that both improved when using a fully automated preselection approach to measure MTV and TLG (using SUV≥4.0 and volume≥3ml).

Ilyas et al. [10] compared three MTV segmentation methods (SUV≥2.5, 41%MAX and PERCIST) in patients with DLBCL and concluded that data-driven optimal cutoff values for separation of patients into a good and a poor prognosis group were largely dependent on the method used, but these data-driven cutoff values had comparable prognostic accuracy. In a subset of 50 patients evaluated by two observers, they found that interobserver reliability was excellent (ICC > 0.98). They further reported a mean analysis time ranging between 2.7 and 6.2 min for the 3 methods [10]. The data-analysis in our study took more time, possibly due to less experience of the observers with the software and the datasheets that had to be completed, which was not included in the time per patient reported in Ilyas study. Yet, also in our study, we found that when total metabolic tumor volume was derived using the preselection and when unwanted normal tissue uptake could be removed and missed lesions could be added by single mouse clicks, the overall processing time was typically less than 5 min. In cases where manual corrections or manual definitions of the VOIs were needed, processing time could well exceed 20 min.

Another important finding in the Ilyas study and our study is that the SUV≥2.5 method showed the highest interobserver reliability. Interestingly though, the observers in our study considered that SUV≥2.5 often overestimated the volume compared with other methods and was almost never chosen as their preferred method on a patient-level.

However, a recent study (partly by the same authors) showed that a slightly higher threshold (SUV≥4.0) outperformed the SUV≥2.5 in terms of success rate [23].

A recent phantom and patient study in primary mediastinal B cell lymphoma that compared four different MTV methods found that SUV≥2.5 resulted in an overestimation, particularly at high SUV values and 41%MAX underestimated MTV when there were high levels of heterogeneity [24].

In a publication by Meignan et al. [12], two observers used two percentage-based methods for MTV assessment in DLBCL (41%MAX and a variable SUVmax threshold that visually resulted in optimal segmentations). They found substantial reliability of 0.99 for the 41%MAX threshold and poor reliability of 0.86 for the variable percentage of SUVmax according to Lin’s concordance correlation coefficient. This study also suggests that reliability decreased with an increasing level of user interaction.

Based on the ratings of individual lesions it could be argued that no single semi-automated segmentation method performed well for every patient and within every lesion of that patient. Lymphoma sites can be difficult to segment because of heterogeneity within and between lesions. Some patients have many lesions, making it almost impossible to delineate each lesion. Besides that, it should be noted that a visual check of the generated segmentation by an experienced nuclear medicine physician or radiologist is necessary if a semi-automated method is applied, as was illustrated by the outliers in this pilot study. For example, patient 10 showed a large difference between the three workflows (Figs. 1 to 3). It appeared that the decision whether the liver was involved or not was the main reason for the large differences in the assessments. Both the observers and the NM physicians did not agree on the question of whether the liver was involved or not. In clinical practice, access to additional clinical information (*e.g.,* physical examination or lab results) may help to support the decision whether a site is involved or not. This situation illustrates the importance of the development of clear clinical criteria, definitions, and guidelines for lesion selection in PET/CT studies of patients with different lymphoma types [25].

We also compared the results of the observers (who were clinicians, but not NM physicians) before and after the check of the NM physician. It appeared that only small lesions were added, and in a few patients, physiological uptake was erroneously included in MTV, again supporting the need for checking of results by a NM physician.

This study has strengths and limitations that should be taken into account when interpreting the results. First, we deliberately selected patients with a large variation in number and size of lesions. This might be a strength because it represents examples of different challenges that can occur when analyzing MTV in lymphoma, but this could give a higher prevalence of difficult cases compared with the general DLBCL cohort. However, according to the three experienced nuclear medicine physicians, the dataset was representative of a general DLBCL cohort, even though we selected a relatively small number of patients.

Another strength is the comparison of different workflows for MTV and TLG assessment and their impact on interobserver reliability. Most studies acknowledge the difficulties in the assessment of multiple lymphoma lesions. Some used boxes or VOIs to constrain individual tumors [6, 8, 12], or limited segmentation to a representative maximum of 5 lymphoma lesions [26], but none of these studies compared such strategies with another workflow.

A limitation is the dependency of the ICC values on the range of MTV values in the population [21]. This is present in other MTV studies as well and hampers comparability of ICCs within and between studies. Therefore, we also presented CoVs and Bland-Altman plots which are not dependent on the variability of MTV values among patients.

Finally, a preselection strategy as suggested in this study is not yet widely available in other commercially available (clinical) software tools but could be implemented relatively easily after validation in a larger patient cohort.

Future research should focus on the comparison of a preselection strategy in a larger patient cohort with different segmentation methods, their success rates, and the effect on the prognostic value of MTV and TLG measurements. A possible solution for the problem that none of the methods will be satisfactory in each patient and for each lesion could be the use of a MV approach, which should be investigated further. In addition, the effect of reconstruction settings, different uptake times, and effect of adding small lesions on the accuracy of MTV and TLG measurements should be addressed.

## Conclusions

A semi-automated workflow based on individual lesion selection (Workflow A) is not recommended, because of the large differences observed in lesion selection. Using a fully automated preselection (SUV≥4.0 and volume≥3ml, Workflow B) of lesions improved interobserver reliability and ease of use of MTV and TLG assessment in DLBCL patients. Subsequent manual modification (Workflow C) is necessary for some patients, but this reduced interobserver reliability which may need to be balanced against any potential improvement of prognostic accuracy.

## Electronic Supplementary Material

ESM 1 (DOCX 1854 kb)
