# Supplementary material for: Optimizing Workflows for Fast and Reliable Metabolic Tumor Volume Measurements in Diffuse Large B Cell Lymphoma
Source: Mol Imaging Biol. 2020 Jan 28;22(4):1102–10. doi: 10.1007/s11307-020-01474-z (PMC7343740; doi:10.1007/s11307-020-01474-z)

**SUPPLEMENTARY MATERIAL**

***Concise and informative title:***

Optimizing workflows for fast and reliable Metabolic Tumor Volume measurements in Diffuse Large B-cell Lymphoma

***Authors:***

Coreline N. Burggraaff^1^, Fareen Rahman^2^, Isabelle Kaßner^3^, Simone Pieplenbosch^1^, Sally F. Barrington^4^, Yvonne W.S. Jauw^1,5^, Gerben J.C. Zwezerijnen^5^, Stefan Müller^3^, Otto S. Hoekstra^5^, Josée M. Zijlstra^1^, Henrica C.W. De Vet^6^, Ronald Boellaard^5^

***Shortened title:***

Workflow optimization of MTV in DLBCL

***Journal:***

Molecular Imaging and Biology

***Manuscript category:***

Original article

***Affiliations:***

On behalf of the PETRA Consortium

^1^Department of Hematology, Amsterdam UMC, Vrije Universiteit Amsterdam, Cancer Center Amsterdam, The Netherlands ([c.burggraaff@amsterdamumc.nl](mailto:c.burggraaff@amsterdamumc.nl); [s.pieplenbosch@amsterdamumc.nl](mailto:s.pieplenbosch@amsterdamumc.nl); [yws.jauw@amsterdamumc.nl](mailto:yws.jauw@amsterdamumc.nl); [j.zijlstra@amsterdamumc.nl](mailto:j.zijlstra@amsterdamumc.nl))

^2^Department of Clinical Oncology, Guy’s and St Thomas’ NHS Foundation Trust, Guys Cancer, London Bridge, United Kingdom ([fareen@doctors.org.uk](mailto:fareen@doctors.org.uk))

^3^Department of Nuclear Medicine, University Hospital Essen, University of Duisburg-Essen, Germany

(isabelle.k87@googlemail.com; [stefan.mueller@uni-due.de](mailto:stefan.mueller@uni-due.de))

^4^King’s College London and Guy’s and St Thomas’ PET Centre, School of Biomedical Engineering and Imaging Sciences, King’s College London, King’s Health Partners, London, United Kingdom ([sally.barrington@kcl.ac.uk](mailto:sally.barrington@kcl.ac.uk))

^5^Department of Radiology and Nuclear Medicine, Amsterdam UMC, Vrije Universiteit Amsterdam, Cancer Center Amsterdam, The Netherlands ([yws.jauw@amsterdamumc.nl](mailto:yws.jauw@amsterdamumc.nl); [g.zwezerijnen@amsterdamumc.n](mailto:g.zwezerijnen@amsterdamumc.n)l; [os.hoekstra@amsterdamumc.nl](mailto:os.hoekstra@amsterdamumc.nl); [r.boellaard@amsterdamumc.nl](mailto:r.boellaard@amsterdamumc.nl))

^6^Department of Epidemiology and Biostatistics, Amsterdam UMC, Vrije Universiteit Amsterdam, Amsterdam Public Health research institute, The Netherlands ([hcw.devet@amsterdamumc.nl](mailto:hcw.devet@amsterdamumc.nl))

***Corresponding author: First author:***

Prof. Ronald Boellaard, PhD Coreline N. Burggraaff, MD, PhD candidate

r.boellaard@amsterdamumc.nl c.burggraaff@amsterdamumc.nl

ORCID: 0000-0001-5863-6528

Address: Department of Radiology and Nuclear Medicine

Amsterdam UMC, Vrije Universiteit Amsterdam,

De Boelelaan 1117

1081HV Amsterdam

Phone: +31(0)2044449638

**SUPPLEMENTAL FIGURE 1**
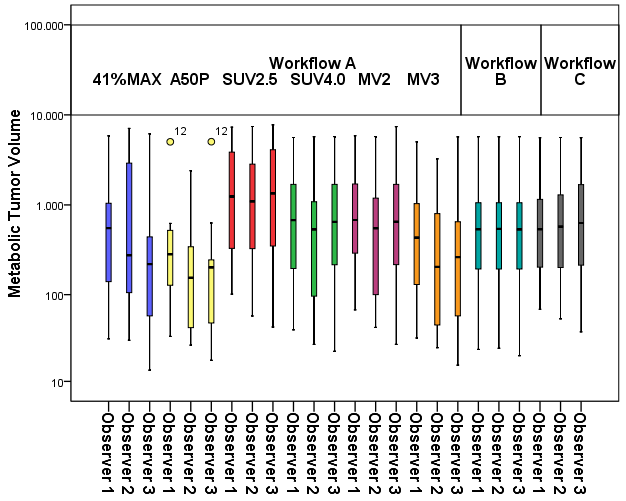


**SUPPLEMENTAL FIGURE 2**


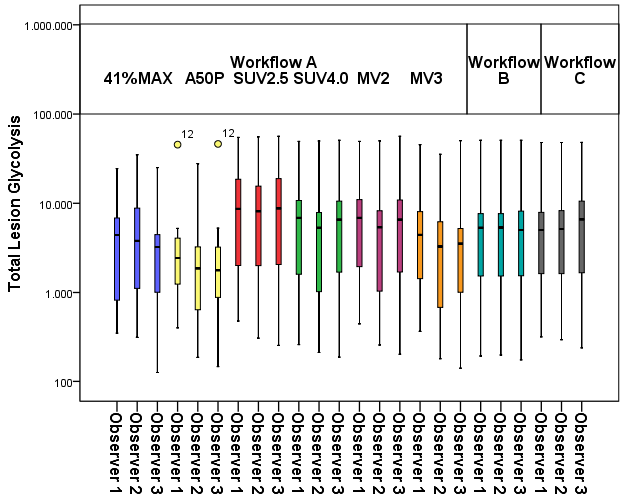

Supplement: Supplementary file 1 — (DOCX 1854 kb) [file 11307_2020_1474_MOESM1_ESM.docx]
